# Investigation of Mother-to-Child Transmission of Hepatitis B in Yinchuan, China: Cross-Sectional Survey Study

**DOI:** 10.2196/60021

**Published:** 2024-09-04

**Authors:** Jie Gu, Yiyuan Xu, Jiao Yuan, Yuxiang Chen, Jingxia Luo, Cui Guo, Guanbin Zhang

**Affiliations:** 1 Department of Neonatology Yinchuan Women and Children Healthcare Hospital Yinchun China; 2 Fujian CapitalBio Medical Laboratory Fuzhou China; 3 Beijing Ganjiang Biotechnology Beijing China; 4 Department of Laboratory Medicine Fujian Medical University Fuzhou China; 5 Institute of Precision Medicine Fujian Medical University Fuzhou China

**Keywords:** hepatitis B virus, mother-to-child transmission, antiviral therapy, immunization failure, cord blood

## Abstract

**Background:**

Hepatitis B poses a significant global public health challenge, with mother-to-child transmission (MTCT) being the primary method of hepatitis B virus (HBV) transmission. The prevalence of HBV infection in China is the highest in Asia, and it carries the greatest burden globally.

**Objective:**

This study aims to critically evaluate the existing local strategies for preventing MTCT and the proposed potential enhancements by analyzing the prevalence of hepatitis B among pregnant women and their neonates in Yinchuan.

**Methods:**

From January 2017 to December 2021, 37,557 prenatal screening records were collected. Among them, 947 pregnant women who tested positive for hepatitis B surface antigen (HBsAg) near delivery and their 960 neonates were included in an HBV-exposed group, while 29 pregnant women who tested negative and their 30 neonates were included in an HBV-nonexposed group. HBV markers in maternal peripheral blood and neonatal cord blood were analyzed using the least absolute shrinkage and selection operator (LASSO) regression, logistic regression, chi-square test, *t*-test, and *U*-test. Additionally, to further evaluate the diagnostic value of HBsAg positivity in cord blood, we conducted an additional follow-up study on 103 infants who tested positive for HBsAg in their cord blood.

**Results:**

The prevalence of HBV among pregnant women was 2.5% (947/37,557), with a declining trend every year (*χ*²_4_=19.7; *P*=.001). From 2018 to 2020, only 33.0% (35/106) of eligible pregnant women received antiviral medication treatment. Using LASSO regression to screen risk factors correlated with HBsAg positivity in cord blood (when log [λ] reached a minimum value of –5.02), 5 variables with nonzero coefficients were selected, including maternal hepatitis B e-antigen (HBeAg) status, maternal hepatitis B core antibody (HBcAb) status, maternal HBV DNA load, delivery method, and neonatal birth weight. Through univariate and multivariate logistic regression, delivery by cesarean section (adjusted odds ratio [aOR] 0.52, 95% CI 0.31-0.87), maternal HBeAg positivity (aOR 2.05, 95% CI 1.27-3.33), low maternal viral load (aOR 2.69, 95% CI 1.33-5.46), and high maternal viral load (aOR 2.69, 95% CI 1.32-5.51) were found to be strongly associated with cord blood HBsAg positivity. In the additional follow-up study, 61 infants successfully completed the follow-up, and only 2 were found to be infected with HBV. The mothers of both these infants had detectable HBV DNA levels and should have received standard antiviral therapy. The results of the hepatitis B surface antibody (HBsAb) positivity rate and titer test indicated a gradual decline in the immunity of vaccinated infants as the interval after vaccination increased.

**Conclusions:**

The clinical relevance of HBV marker detection in cord blood is restricted within the current prevention measures for MTCT. There is an emphasis on the significance of public education regarding hepatitis B and the reinforcement of postnatal follow-up for the prevention of MTCT.

## Introduction

Hepatitis B is a significant global public health challenge. According to the World Health Organization (WHO), viral hepatitis ranked second (only to COVID-19) among communicable diseases as a cause of death in 2022. Notably, it caused 1.3 million deaths, with hepatitis B accounting for 83% of deaths, imposing a significant disease burden globally [1]. The predominant mode of transmission for hepatitis B virus (HBV) is mother-to-child transmission (MTCT) [2]. The estimated global population of women at reproductive age with chronic hepatitis B is approximately 75 million, thereby exposing their offspring to the risk of HBV infection [3]. Annually, nearly 2 million children younger than 5 years are infected with HBV [4]. Without effective intervention, the risk of HBV MTCT can escalate to as high as 90% in the Asian region [4,5]. Worse still, these children face a 95% risk of developing chronic hepatitis B in their later years, accompanied by a 40% lifetime risk of developing cirrhosis and hepatocellular carcinoma [3]. The WHO has appealed to eliminate hepatitis B as a threat to public health security by 2030, which can be accomplished by implementing preventive measures to interrupt HBV MTCT and maintaining a prevalence rate below 0.1% among children younger than 5 years [6]. The prevalence of hepatitis B among children in Southeast Asia and the Western Pacific has decreased due to vaccination programs, irrespective of the specific vaccination schedule [7]. In 2019, the global prevalence of HBV among children younger than 5 years was reported to be 1.3% [4]. Significant efforts are still required to bridge the existing gap to accomplish the WHO’s goal of eliminating hepatitis B.

China has the highest prevalence of HBV infection in Asia and bears the greatest burden globally, with approximately 70 million carriers and a prevalence rate of 5%-6% [8-10]. The prevalence of HBV infection in China has significantly decreased in recent years due to the implementation of policies such as the provision of free prenatal hepatitis B screening, antiviral therapy for pregnant women with high viral load, hepatitis B vaccination for neonates, and follow-up care for exposed infants after HBV vaccination. It is expected that China will fully achieve the target set by the WHO by 2030 [10-13]. Moreover, the feasibility and effectiveness of the aforementioned hepatitis B preventive measures have been demonstrated in a nationwide study across hospitals at various levels and economic development areas [14]. However, it is worth noting that although the prevalence of hepatitis B among pregnant women in various regions of China generally decreased from 2015 to 2020, regional disparities in disease burden still exist [15]. The prevalence is higher in the eastern and western regions as well as rural areas when compared to the prevalence in the central region and urban areas [12,15-17]. The potential factors contributing to this phenomenon include maternal age, educational attainment, employment status, ethnicity, pregnancy history, and regional economic differences [2,16,17]. To achieve the goal of eliminating hepatitis B, the strategies for preventing MTCT of hepatitis B should be tailored to the local context. Yinchuan City is located in the inland northwest of China, belonging to Ningxia Hui Autonomous Region, which has a moderate prevalence of HBV infection [9,15]. In recent years, there have been rare reports on the prevalence of HBV among pregnant women in Yinchuan, as well as their antiviral therapy status and the follow-up results of their delivered infants. Therefore, it is imperative to enhance regional epidemiological investigations on HBV MTCT in this area to provide valuable guidance for the clinical implementation of prevention measures.

Yinchuan Women and Children Healthcare Hospital (YWCHH) is a prominent local institution that plays a representative role in providing maternal and child health care services. This study aimed to explore the prevalence of hepatitis B among pregnant women in Yinchuan and assess their antiviral medication usage by analyzing hospitalization data and follow-up results at YWCHH. It also investigated the correlation between the detection of HBV markers in cord blood and infant immunization failure as well as immune status in infants after vaccination. These assessments are anticipated to offer valuable insights for developing more effective measures to prevent MTCT.

## Methods

### Study Design and Data Collection

From January 2017 to December 2021, we conducted a retrospective cross-sectional study at YWCHH and its Yuehai branch, involving a total of 37,557 pregnant women who received delivery services at these medical institutions. The pregnant women and their neonates were categorized into an HBV-exposed group and an HBV-nonexposed group, based on the detection results of hepatitis B surface antigen (HBsAg) in maternal peripheral blood. The hospital’s electronic medical records were used to gather information regarding the pregnant women and their neonates. The collected data included maternal age, gestational week, mode of delivery, medication history, neonatal gender, birth weight, and HBV marker test results of both the mother and neonate. HBV makers included HBsAg, hepatitis B surface antibody (HBsAb), hepatitis B e-antigen (HBeAg), hepatitis B e-antibody (HBeAb), hepatitis B core antibody (HBcAb), and HBV DNA.

Based on these assessments, a significant correlation was observed between positive neonatal cord blood HBsAg and positive maternal HBV markers, including HBeAg and HBV DNA. To evaluate the diagnostic value of HBsAg positivity in cord blood, we initiated an additional follow-up study from June 2021 to August 2022, involving 103 infants in the HBV-exposed group who tested positive for HBsAg in cord blood. We reached out to the parents of these infants via telephone and invited them to bring their infants to the hospital for blood HBV marker testing. This additional study was not aimed at establishing a cohort for longitudinal tracking but rather served to enhance our understanding of HBsAg positivity in cord blood by providing a more comprehensive characterization.

Although a follow-up period was included, this study should be classified as a cross-sectional study owing to the limited number of infants observed with positive HBsAg in cord blood, in accordance with the original investigation.

The hospital electronic medical records did not include data on the maternal delivery method in 2017, as well as maternal medication history for both 2017 and 2021. Consequently, our analysis can only be conducted based on the available data set. It is important to highlight that in China, HBV DNA testing is not mandatory among HBsAg-positive pregnant women and their neonates. Therefore, participants who declined to undergo HBV DNA testing were marked as “not tested” in this study.

### Stratification by HBV Exposure

The inclusion criteria for the HBV-exposed group were: (1) pregnant women who tested positive for HBsAg during prenatal screening and (2) their partners were not infected with HBV. The exclusion criteria for the HBV-exposed group were: (1) pregnant women who declined to have their neonates’ cord blood tested; (2) pregnant women who did not undergo HBV testing either within 1 month prior to delivery or within 1 week postpartum; and (3) individuals who did not undergo quantitative HBV marker testing. This study enrolled 947 pregnant women and 960 neonates (including 13 pairs of twins) in the HBV-exposed group.

Owing to restrictions imposed by China’s national medical insurance policy, HBsAg-negative mothers’ neonates are precluded from undergoing HBV marker testing on cord blood. However, when treatment for neonatal jaundice or anemia is required, peripheral blood HBV markers of the neonates are screened, which can partially reflect the hepatitis B infection and immune status of the infants. Therefore, we included HBsAg-negative mothers and their neonates who had undergone peripheral blood HBV marker testing in the HBV-nonexposed group for analysis. The inclusion criteria for the HBV-nonexposed group were: (1) pregnant women who tested negative for HBsAg during prenatal screening and (2) their partners were not infected with HBV. The exclusion criteria for the HBV-nonexposed group were that pregnant women and neonates did not undergo quantitative HBV marker testing. After screening all the data from 2021, we included a total of 29 eligible pregnant women and 30 neonates (including 1 pair of twins) in our study.

### Sample Collection

Maternal peripheral blood samples were collected either within 1 month prior to delivery or within 1 week postpartum. Neonatal cord blood samples were collected via needle aspiration after surface rinsing of the umbilical cord with normal saline. The peripheral blood of infants in the HBV-exposed group was collected by scheduling an appointment for them to return to the hospital after the telephone follow-up. The blood samples were collected by trained medical personnel. The volume of each blood sample was 2-3 mL, and the serum was separated within 2 hours of collection. It was then stored at –20 ℃ until HBV marker detection, which was completed within 24 hours.

### Detection of HBV Markers

From 2017 to 2021, a series of chemiluminescence kits (Autobio) were used in YWCHH for the detection of HBsAg, HBsAb, HBeAg, HBeAb, and HBcAb. The positive thresholds were HBsAg ≥0.05 IU/mL, HBsAb ≥10 IU/L, HBeAg ≥0.1 PEIU/mL, HBeAb ≥0.4 PEIU/mL, and HBcAb ≥0.7 PEIU/mL. The Yuehai branch of YWCHH also used Autobio reagents for detection from 2017 to 2018. However, the detection method was switched to electrochemiluminescence kits (Roche Diagnostics) from 2019 to 2021. The positive thresholds were HBsAg ≥1 cutoff index (COI), HBsAb ≥10 IU/L, HBeAg ≥1 COI, HBeAb ≤1 COI, and HBcAb ≤1 COI.

HBV DNA load was tested using the HBV nucleic acid detection kit (PCR-Fluorescent Probe Method; Daan Gene). A sample with HBV DNA concentration greater than 1000 IU/mL was reported as positive.

### Active-Passive Immunoprophylaxis in Neonates

According to the national immunization program, all neonates delivered by HBsAg-positive pregnant women receive 1 dose of hepatitis B hyperimmune globulin (HBIG) (100 IU; Hualan Bio) and the first dose of hepatitis B vaccine (HepB) (10 μg; Aim Honesty) within 24 hours after birth. Subsequently, they receive repeat doses of HepB when they reach 1 month and 6 months of age.

### Statistical Analysis

For descriptive statistics, continuous variables have been presented as mean (SD), while categorical variables have been expressed as number (percentage). Significant differences between groups were analyzed by employing suitable statistical testing methods, including the independent samples *t*-test, chi-square test (Pearson chi-square test or Fisher exact test), and Mann-Whitney *U* test. A least absolute shrinkage and selection operator (LASSO) binary logistic regression model was built to screen risk factors correlated with HBsAg positivity in cord blood. Then, univariate logistic regression analyses were conducted to demonstrate substantial correlation. Subsequently, these factors were incorporated into a multivariate logistic regression model to assess their combined impact on cord blood HBsAg positivity. The results of logistic regression analysis are expressed as odds ratio (OR) (95% CI).

All *P* values were 2-sided, and the threshold for statistical significance was set at *P*<.05. The calculation and image drawing for the LASSO regression analysis were performed using the “glmnet” package and “ggplot2” package in R software (version 4.4.0). The rest of the statistical analyses were performed with IBM SPSS Statistics 26.0 (IBM Corp).

### Definition

According to the evaluation of immune response in infants after vaccination in the guidelines [18], the presence of HBsAg in peripheral blood during follow-up indicates immunization failure. Conversely, the presence of HBsAb and absence of HBsAg indicates successful immunization. The absence of both HBsAg and HBsAb indicates no response to immunization.

According to the guidelines [18-20], patients with HBV DNA concentration exceeding 200,000 IU/mL are defined as high viral load patients, while those with HBV DNA concentration between 1000 IU/mL and 200,000 IU/mL are defined as low viral load patients.

### Ethical Considerations

The study adhered to the requirements of the revised Declaration of Helsinki in 2013 and received approval from the Ethics Committee of YWCHH (ID: Municipal Maternal and Child Ethics No. 2021-01). All research data have been anonymized to ensure that the privacy of the subjects is fully protected. Pregnant women were verbally informed during delivery in medical institutions, and their anonymous data collected before delivery and at the birth of newborns were included in the study. Given that the data collection and extraction process strictly adhered to the principle of anonymity, the Ethics Committee of YWCHH approved this study with an exemption from individual informed consent (ID: Municipal Maternal and Child Ethics No. 2021-02). For infants participating in the follow-up study, we provided an informed consent notice to the infants’ legal guardians and obtained verbal informed consent from them. In this study, participants did not receive direct financial rewards. However, infants undergoing follow-up can obtain a complimentary serological hepatitis B marker test.

## Results

### HBV Infection Status in Pregnant Women

**Figure 1:**
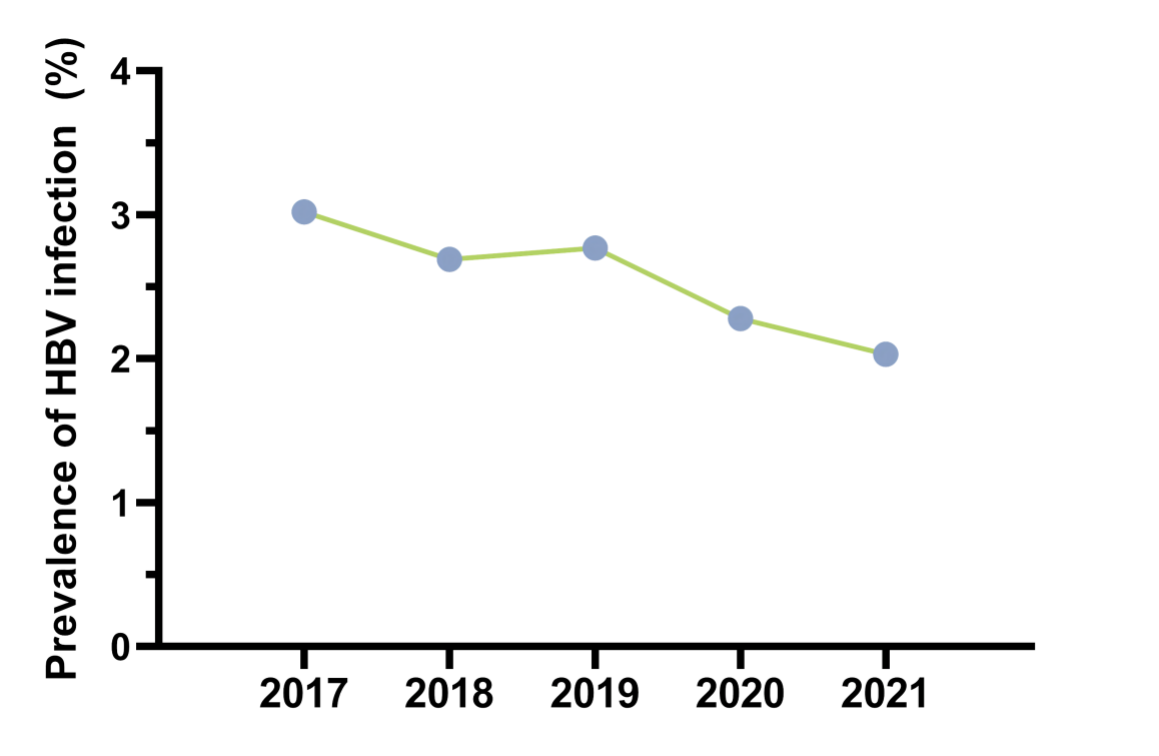
Declining hepatitis B virus (HBV) infection rate among pregnant women who attended Yinchuan Women and Children Healthcare Hospital from 2017 to 2021.

The total HBV prevalence rate among pregnant women visiting the hospital from 2017 to 2021 was 2.5% (947/37,557). As shown in Figure 1, the annual prevalence rates were 3.0% (193/6400), 2.7% (175/6509), 2.8% (222/8019), 2.3% (178/7792), and 2.0% (179/8837) for 2017, 2018, 2019, 2020, and 2021, respectively. A significant disparity was observed in the prevalence of pregnant women across each year, indicating a notable declining trend over time (*χ*²_4_=19.7; *P*=.001).

### Participant Characteristics

The study enrolled 947 pregnant women and 960 neonates (including 13 pairs of twins) in the HBV-exposed group, and 29 pregnant women and 30 neonates (including 1 pair of twins) in the HBV-nonexposed group. The characteristics of pregnant women and neonates between these 2 groups are shown in Tables 1 and 2, respectively. There were no statistically significant differences between the 2 groups in terms of maternal age, delivery method, neonatal birth weight, and neonatal gender. In the HBV-exposed group, HBeAg positivity was detected in 29.4% (278/947) of cases. Additionally, 7.5% (71/947) of pregnant women had a high viral load, 7.4% (70/947) had a low viral load, 46.6% (440/947) had negative findings, and 38.7% (367/947) refused to undergo HBV DNA testing. In the HBV-nonexposed group, all pregnant women tested negative for HBV infection, with 62.1% (18/29) testing positive for HBsAb and 37.9% (11/29) testing negative. The presence of HBV markers in cord blood was only observed in neonates from the HBV-exposed group, with 10.4% (103/960) testing positive for HBsAg, 22.1% (222/960) testing positive for HBeAg, and 5.0% (48/960) testing positive for both markers. Additionally, 0.2% (2/960) of neonates had a high viral load, 1.0% (10/960) had a low viral load, 92.2% (885/960) had negative findings, and 6.6% (63/960) did not undergo HBV DNA testing. Additionally, HBV DNA testing was not conducted on pregnant women and neonates in the nonexposed group owing to the absence of any indications.

**Table 1:** Maternal characteristics in the hepatitis B virus–exposed and nonexposed groups (N=976).

Characteristic			HBV^a^-exposed group (n=947)	HBV-nonexposed group (n=29)	*P* value
Age (years), mean (SD)			31.1 (4.5)	31.9 (4.6)	.34
Gestational week (weeks), mean (SD)			38.8 (1.7)	37.3 (2.8)	.008
**Delivery method, n (%)**					.28
	Vaginal	415 (43.8)		13 (44.8)	
	Cesarean	339 (35.8)		16 (55.2)	
	Not recorded	193 (20.4)		0 (0)	
**HBsAb^b^ status, n (%)**					<.001
	Positive	21 (2.2)		18 (62.1)	
	Negative	926 (97.8)		11 (37.9)	
**HBeAg^c^ status, n (%)**					.001
	Positive	278 (29.4)		0 (0)	
	Negative	669 (70.6)		29 (100)	
**HBeAb^d^ status, n (%)**					<.001
	Positive	614 (64.8)		0 (0)	
	Negative	333 (35.2)		29 (100)	
**HBcAb^e^ status, n (%)**					<.001
	Positive	933 (98.5)		0 (0)	
	Negative	14 (1.5)		29 (100)	
**HBV DNA load, n (%)**					—^f^
	High viral load (>2×10^5^ IU/mL)	71 (7.5)		0 (0)	
	Low viral load (≥1×10^3^ and ≤2×10^5^ IU/mL)	70 (7.4)		0 (0)	
	Negative (<1×10^3^ IU/mL)	440 (46.5)		0 (0)	
	Not tested	366 (38.6)		29 (100)	

^a^HBV: hepatitis B virus.

^b^HBsAb: hepatitis B surface antibody.

^c^HBeAg: hepatitis B e-antigen.

^d^HBeAb: hepatitis B e-antibody.

^e^HBcAb: hepatitis B core antibody.

^f^Not applicable.

**Table 2:** Neonatal characteristics in the hepatitis B virus–exposed and nonexposed groups (N=990).

Characteristic		HBV^a^-exposed group (n=960)	HBV-nonexposed group (n=30)	*P* value
Birth weight (g), mean (SD)		3276.0 (512.2)	3047.8 (699.0)	.09
**Gender, n (%)**				.30
	Male	477 (49.7)	18 (60.0)	
	Female	483 (50.3)	12 (40.0)	
**HBsAg^b^ status, n (%)**				.06
	Positive	103 (10.4)	0 (0)	
	Negative	857 (89.3)	30 (100)	
**HBeAg^c^ status, n (%)**				.001
	Positive	222 (23.1)	0 (0)	
	Negative	738 (76.9)	30 (100)	
**HBeAb^d^ status, n (%)**				<.001
	Positive	633 (65.9)	0 (0)	
	Negative	327 (34.1)	30 (100)	
**HBcAb^e^ status, n (%)**				<.001
	Positive	946 (98.5)	0 (0)	
	Negative	14 (1.5)	30 (100)	
**HBV DNA load, n (%)**				—^f^
	High viral load (>2×10^5^ IU/mL)	2 (0.2)	0 (0)	
	Low viral load (≥1×10^3^ and ≤2×10^5^ IU/mL)	10 (1.0)	0 (0)	
	Negative (<1×10^3^ IU/mL)	885 (92.2)	0 (0)	
	Not tested	63 (6.6)	30 (100)	

^a^HBV: hepatitis B virus.

^b^HBsAg: hepatitis B surface antigen.

^c^HBeAg: hepatitis B e-antigen.

^d^HBeAb: hepatitis B e-antibody.

^e^HBcAb: hepatitis B core antibody.

^f^Not applicable.

Of the 103 neonates in the exposed group with HBsAg-positive cord blood, 61 completed the follow-up, with a success rate of 59.2%. The reasons for follow-up failure included the inability to establish contact with parents (wrong contact information or refusal to answer phone calls) and refusal to visit the hospital for peripheral blood collection from their neonates (rejected the sampling request, already self-tested, or relocated from a local area).

### HBV Antiviral Therapy in Pregnant Women From 2018 to 2020

**Figure 2:**
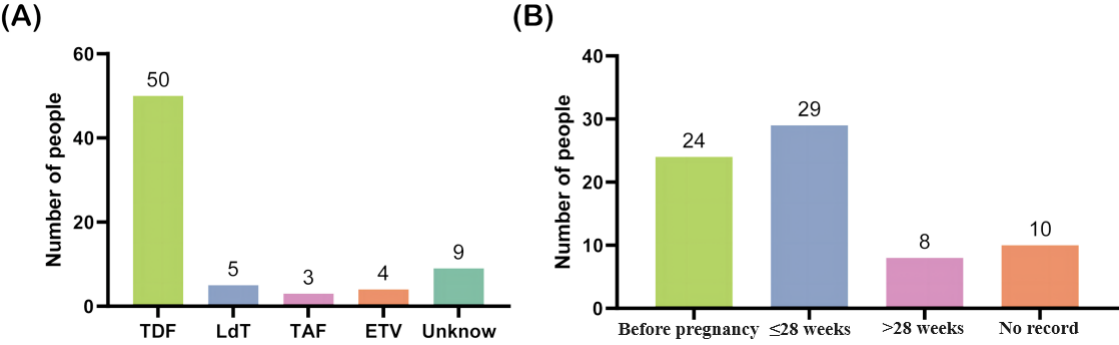
Statistics on antiviral treatment during pregnancy from 2018 to 2020, as documented in the hospital’s electronic medical records. (A) Statistics on antiviral drugs used by pregnant women show that TDF was the most commonly used. (B) Statistics on the timing of antiviral therapy initiation among pregnant women show that the majority of them had started antiviral therapy by or before the 28th week of gestation. ETV: entecavir; LdT: telbivudine; TAF: tenofovir alafenamide fumarate; TDF: tenofovir disoproxil fumarate.

Nucleotide analogs (NAs) are recommended for HBV antiviral therapy in pregnant women with HBV DNA loads exceeding 200,000 IU/mL [18-20]. Statistics on antiviral therapy during the period of 2018 to 2020 are available. Among a total of 575 HBsAg-positive pregnant women, 71 (12.4%) received NA therapy during pregnancy. The most used NAs were tenofovir disoproxil fumarate (TDF) (50/71, 70%), telbivudine (LdT) (5/71, 7%), entecavir (ETV) (4/71, 6%), and tenofovir alafenamide fumarate (TAF) (3/71, 4%). However, medication records for 13% (9/71) of the pregnant women were unknown (Figure 2A). The therapy was initiated prior to pregnancy in 24 cases (34%), with it being initiated before reaching 28 weeks of gestation in 29 cases (41%) and initiated after surpassing 28 weeks of gestation in 8 cases (11%). Additionally, the initiation time was not recorded in 10 cases (14%) (Figure 2B).

The HBV DNA test results of pregnant women undergoing antiviral therapy at the time of delivery are shown in Table 3. Among the 71 cases, 20 (28%) had negative findings, 17 (24%) had a low viral load, 4 (6%) had a high viral load, and 30 (42%) did not undergo testing. The results indicated that although a higher proportion of pregnant women in the group with earlier antiviral therapy initiation had negative findings or a low HBV DNA load, there were no statistically significant differences across groups (*P*=.21).

**Table 3:** Hepatitis B virus DNA load near delivery in pregnant women undergoing antiviral therapy (N=71).

Timing of antiviral therapy initiation	HBV^a^ DNA load, n (%)				*P* value
	Negative (n=20, 28%)	Low viral load (n=17, 24%)	High viral load (n=4, 6%)	Unknown (n=30, 42%)	
Before pregnancy	10 (50)	3 (18)	1 (25)	10 (33)	.21
Before 28 weeks of gestation	5 (25)	9 (53)	2 (50)	13 (43)	
After 28 weeks of gestation	2 (10)	1 (6)	1 (25)	4 (13)	
No record	3 (15)	4 (23)	0 (0)	3 (10)	

^a^HBV: hepatitis B virus.

It is noteworthy that a total of 106 pregnant women met the criterion for antiviral medication, but 33% (35/106) of them did not receive any therapy. The aforementioned result was derived from a restricted number of HBV DNA test results, and the actual situation may be even more severe. Owing to the high cost and lack of insurance coverage for the HBV DNA test, many pregnant women decline to undergo this test, thereby hindering the evaluation of their need for antiviral therapy based on their HBV DNA load. In these cases, the presence of HBeAg can be considered as an alternative to HBV DNA for monitoring and evaluating the necessity of antiviral therapy [9,20,21]. If HBeAg positivity is adopted as the criterion, the percentage of pregnant women in this study who should receive antiviral therapy but are not receiving it increases to 67.3% (113/168). This highlights the inadequacy of local medical institutions for effectively implementing antiviral therapy for pregnant women with a high viral load, thereby emphasizing the imperative to further disseminate the knowledge regarding antiviral therapy and facilitate its implementation.

### Comparison of HBV Markers in Maternal and Neonatal Blood Samples

**Figure 3:**
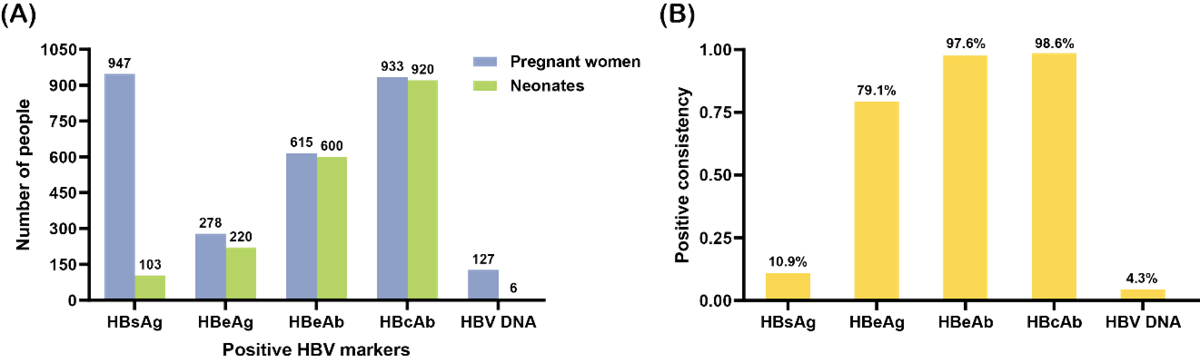
Correlation of HBV markers positive in maternal blood and umbilical cord blood. (A) Comparison of the number of pregnant women with each positive HBV marker and the corresponding positive HBV markers in the cord blood of their neonates. Owing to the limited number of pregnant women (less than 100) with positive HBsAb results, this marker was excluded from the statistics. For 13 pairs of twins, only the test results of the preterm neonates were included in this statistic. (B) The consistency rate of each HBV marker between the pregnant women and neonates. HBcAb: hepatitis B core antibody; HBeAb: hepatitis B e-antibody; HBeAg: hepatitis B e-antigen; HBsAg: hepatitis B surface antigen; HBV: hepatitis B virus.

To investigate the correlation between HBV markers in maternal peripheral blood and those in neonatal cord blood, we included all HBV markers that had more than 100 maternal positive results in our statistical analysis. The number of pregnant women with positive indicators and the number of neonates with positive indicators in cord blood were separately counted. For 13 pairs of twins, this statistic only considered the test results of the preterm neonates, and 14 pregnant women were excluded from the statistics of HBV DNA owing to the lack of detection of HBV DNA in their neonates’ cord blood. The comparison results are presented in Figure 3. The concordance rates of HBsAg, HBeAg, HBeAb, HBcAb, and HBV DNA positivity in maternal peripheral blood and neonatal cord blood were 10.9% (103/947), 79.1% (220/278), 97.6% (600/615), 98.6% (920/933), and 4.3% (6/127), respectively. It is worth noting that all 220 neonates with HBeAg positivity in cord blood were born to HBeAg-positive mothers. Moreover, all 6 neonates, including 1 with a high viral load and 5 with a low viral load in cord blood, were born to pregnant women with high HBV viral loads.

**Figure 4:**
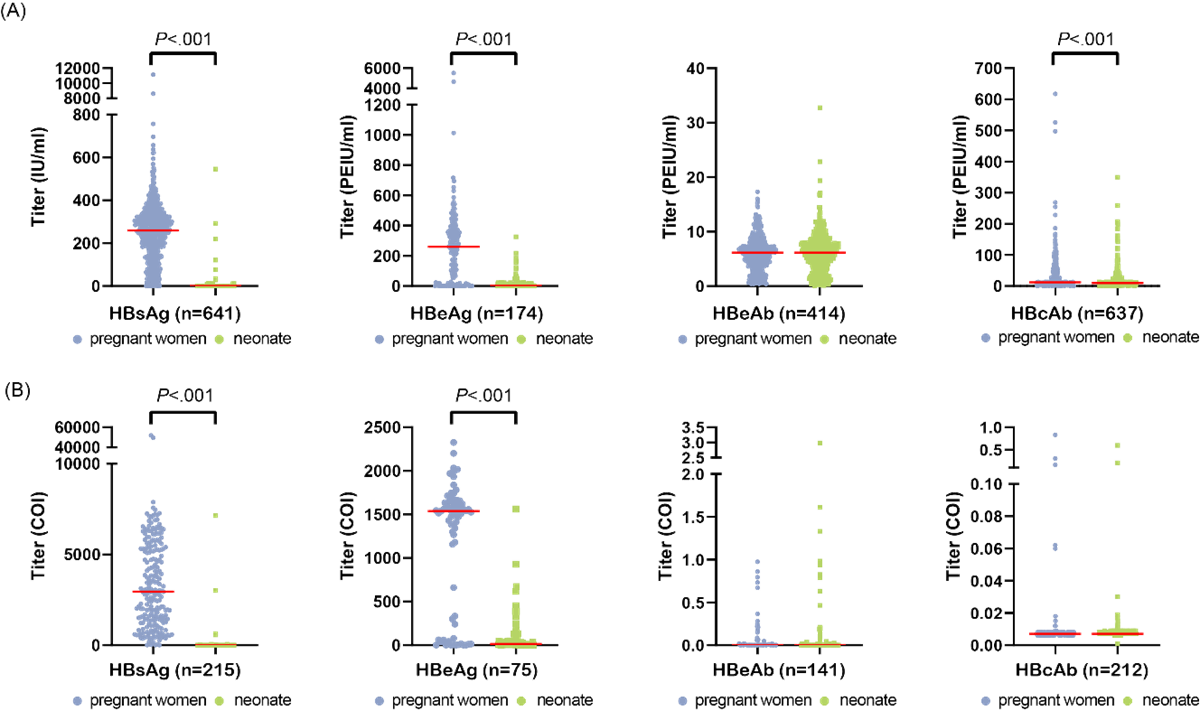
Comparison of titers for various HBV serological markers in maternal peripheral blood and neonatal cord blood. HBsAb was not included in the statistics due to the limited number of pregnant women (less than 100) with positive test results. Additionally, 91 mother-infant pairs with discordant reagent use were excluded from the analysis. (A) The results detected using Autobio reagents. (B) The results detected using Roche reagents. COI: cutoff index; HBcAb: hepatitis B core antibody; HBeAb: hepatitis B e-antibody; HBeAg: hepatitis B e-antigen; HBsAb: hepatitis B surface antibody; HBsAg: hepatitis B surface antigen; HBV: hepatitis B virus.

We analyzed the distribution of titers for these markers in both maternal peripheral blood and neonatal cord blood for further investigation. The data were analyzed separately owing to the use of different testing reagents from 2 manufacturers, Autobio and Roche, with the results presented in Figure 4. It should be noted that 91 mother-infant pairs with discordant reagent use were excluded from the analysis.

When employing quantitative reagents from Autobio for detection, the titers of HBsAg, HBeAg, and HBcAb in neonatal cord blood exhibited a significant reduction compared to maternal peripheral blood (*P*<.001). However, there was no statistically significant difference observed in HBeAb titers (Figure 4A). When employing the semiquantitative reagents from Roche for detection, it was observed that the titers of HBsAg and HBeAg in neonatal cord blood exhibited a significant decrease compared to those in maternal peripheral blood (*P*<.001). However, no notable disparity was found in the titers of HBeAb and HBcAb between them (Figure 4B). The lower sensitivity of Roche reagents may be attributed to the relative value of the sample measurement compared to a reference measurement value.

### Factors Correlated With the Presence of HBsAg in Cord Blood

**Figure 5:**
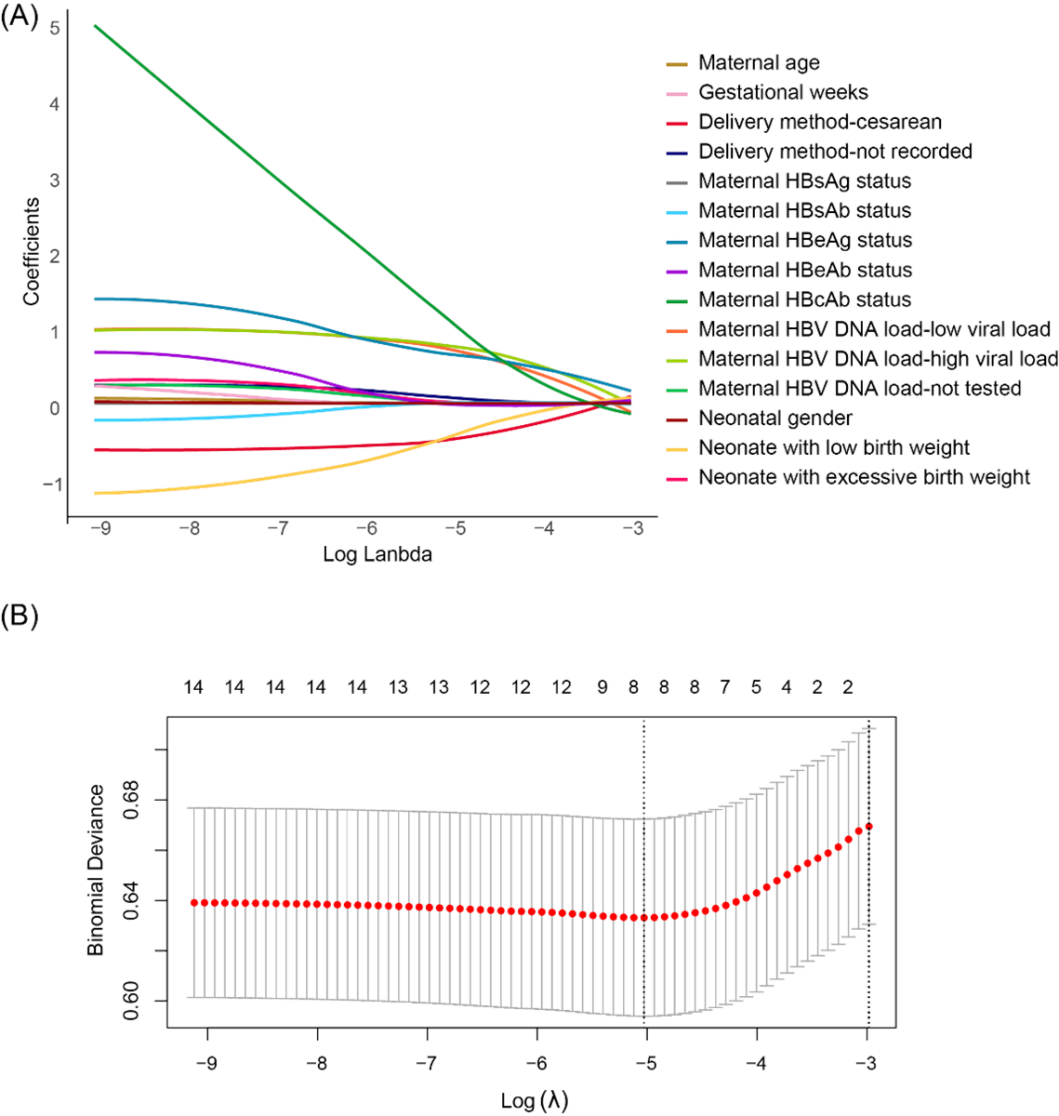
Screening for factors correlated with the presence of HBsAg in cord blood is conducted using least absolute shrinkage and selection operator (LASSO) binary logistic regression. The variables identified include maternal HBeAg status, maternal HBcAb status, maternal HBV DNA load, delivery method, and neonatal birth weight. (A) LASSO regression coefficients for each factor at different penalty parameter values. (B) Ten-fold cross-validation plot for the penalty term. HBcAb: hepatitis B core antibody; HBeAb: hepatitis B e-antibody; HBeAg: hepatitis B e-antigen; HBsAb: hepatitis B surface antibody; HBsAg: hepatitis B surface antigen; HBV: hepatitis B virus.

Maternal relevant variables, along with neonatal gender and birth weight, were included in the LASSO binary logistic regression model for screening. The regression coefficients for each variable under varying values of the penalty parameter are displayed in Figure 5A. After undergoing 10-fold cross-validation, the resulting cross-validation curve has been depicted in Figure 5B. When Log (λ) was –5.028, λ attained its minimum value, demonstrating the optimal fitting performance of the model. The variables obtained by screening included maternal HBeAg status, maternal HBcAb status, maternal HBV DNA load, delivery method, and neonatal birth weight.

The results of the logistic regression analysis for these 5 variables are shown in Table 4. In univariate logistic regression analysis, we conducted an analysis to determine the independent impact of the 5 variables on positive HBsAg results in cord blood. Our findings indicated that both HBcAb status (*P*>.99) and neonatal birth weight (low birth weight: *P*=.07, excessive birth weight: *P*=.89) did not exhibit statistical significance and were subsequently excluded. The remaining 3 variables, including maternal HBeAg status, maternal HBV DNA load, and mode of delivery, were incorporated into the multivariate logistic regression model for examination. Neonates born by cesarean section were less likely to be HBsAg positive than those born vaginally (adjusted OR [aOR] 0.52, 95% CI 0.31-0.87). Maternal HBeAg positivity (aOR 2.05, 95% CI 1.27-3.33), low maternal viral load (aOR 2.69, 95% CI 1.33-5.46), and high maternal viral load (aOR 2.69, 95% CI 1.32-5.51) were identified as risk factors associated with HBsAg positivity in cord blood.

**Table 4:** Logistic regression analysis for the risk factors of the presence of hepatitis B surface antigen in cord blood.

Variable			Univariate analysis			Multivariate analysis	
			OR^a^ (95% CI)		*P* value	aOR^b^ (95% CI)^c^	*P* value
**Delivery method**							
	Cesarean	1.00 (reference)		—^d^		1.00 (reference)	—
	Vaginal	0.48 (0.29-0.79)		.004		0.52 (0.31-0.87)	.01
	No record	1.14 (0.69-1.88)		.61		1.30 (0.77-2.20)	.33
**Maternal HBeAg^e^ status**							
	Negative	1.00 (reference)		—		1.00 (reference)	—
	Positive	2.91 (1.92-4.41)		<.001		2.05 (1.29-3.33)	.003
**Maternal HBV^f^ DNA load**							
	Negative	1.00 (reference)		—		1.00 (reference)	—
	Low viral load	3.53 (1.83-6.80)		<.001		2.69 (1.33-5.46)	.006
	High viral load	4.43 (2.36-8.32)		<.001		2.69 (1.32-5.51)	.007
	Not tested	1.12 (0.68-1.84)		.66		1.19 (0.71-1.99)	.51
**Maternal HBcAb^g^ status**							
	Negative	1.00 (reference)		—		—	—
	Positive	Data anomaly^h^		>.99		—	—
**Neonatal birth weight**							
	Normal	1.00 (reference)		—		—	—
	Low (<2500 g)	0.27 (0.06-1.11)		.07		—	—
	Excessive (>4000 g)	0.94 (0.39-2.25)		.89		—	—

^a^OR: odds ratio.

^b^aOR: adjusted odds ratio.

^c^Adjusted for delivery method, maternal hepatitis B e-antigen status, and maternal hepatitis B virus DNA load.

^d^Not applicable.

^e^HBeAg: hepatitis B e-antigen.

^f^HBV: hepatitis B virus.

^g^HBcAb: hepatitis B core antibody.

^h^As all pregnant women in the nonexposed group tested negative for HBcAb, a complete separation phenomenon occurred, leading to an abnormally high OR value (197,617,633.93, 95% CI 0.00-not displayed).

### Follow-Up Outcomes of Infants With HBsAg Positivity in Cord Blood

To better characterize the infection and evaluate the diagnostic value of testing positive for HBsAg in cord blood, we conducted an additional follow-up study. This additional study was initiated in 2021, following the screening of infants with positive HBsAg in their cord blood from the previous years’ electronic medical records. Consequently, the earlier an infant was born, the longer the interval between their birth and the time of follow-up, ranging from 6 to 64 months.

Among 61 infants with positive HBsAg in cord blood who were successfully followed up, 49 (80%) achieved successful immunization, while 10 (16%) exhibited no response to immunization and 2 (3%) experienced immunization failure. Therefore, the predictive value of cord blood testing for identifying infants at risk of immune failure is limited. The results of peripheral blood detection at follow-up were compared with those of cord blood detection, and the loss rates of HBV markers were as follows: HBsAg, 97% (59/61); HBeAg, 91% (20/22); HBeAb, 100% (33/33); HBcAb, 93% (57/61); and HBV DNA, 67% (2/3).

The relevant data regarding the 2 infants who experienced immunization failure, along with their mothers, have been documented in Table 5. The detection results of HBV serologic markers in these 2 infants at follow-up were consistent with cord blood test results, showing positive results for HBsAg, HBeAg, and HBcAb, and negative results for HBsAb and HBeAb. Additionally, the titers of HBsAg, HBeAg, and HBcAb were significantly higher compared to those observed in cord blood. It is worth noting that despite negative HBV DNA test results and low viral load observed in cord blood screening, both infants exhibited high viral loads during follow-up. This implies that even in the absence of detectable HBV DNA in cord blood, there remains a potential for immunization failure.

**Table 5:** Related characteristics of neonates with immunization failure and their mothers.

Characteristic			Case 1		Case 2
Infant gender			Male		Female
Gestational week			39		38
Mode of delivery			Vaginal		Vaginal
Birth weight (g)			3860		2815
Time of HBIG^a^ (hours)			<24		<24
Time of first HepB^b^ (hours)			<24		<24
Follow-up interval (months)			52		40
Maternal medical treatment			Never		Briefly taken tenofovir
**Maternal HBV^c^ test results at delivery^d^**					
	HBsAg^e^ (IU/mL)	16.67		58.74	
	HBeAg^f^ (PEIU/mL)	402.94		370.85	
	HBV DNA (IU/mL)	99,600,000		244,000,000^g^	
**Infant HBV test results at birth^d^**					
	HBsAg (IU/mL)	0.22		0.88	
	HBeAg (PEIU/mL)	11.60		1.64	
	HBV DNA (IU/mL)	Negative		5460	
**Infant HBV test results at follow-up^d^**					
	HBsAg (IU/mL)	18.99		61.93	
	HBeAg (PEIU/mL)	509.46		119.24	
	HBV DNA (IU/mL)	465,000,000		151,000,000	

^a^HBIG: hepatitis B hyperimmune globulin.

^b^HepB: hepatitis B vaccine.

^c^HBV: hepatitis B virus.

^d^Reference values: HBV DNA (+), ≥1000 IU/mL; HBsAg (+), ≥0.5 IU/mL; HBeAg (+), ≥0.5 PEIU/mL.

^e^HBsAg: hepatitis B surface antigen.

^f^HBeAg: hepatitis B e-antigen.

^g^The pregnant woman did not undergo testing for HBV DNA at the time of delivery; the data were obtained 4 months before delivery.

At the same time, we found that both mothers of infants with immunization failure were eligible for NA therapy, but neither had received effective therapy. One of them, who had never taken NAs since being diagnosed with hepatitis B in the early years, exhibited a high viral load near delivery. Another had a high viral load at 14 weeks of pregnancy and discontinued taking TDF without undergoing subsequent HBV DNA testing after several days. Although we cannot ascertain whether the 2 infants were infected at birth or later in life due to the long follow-up interval (40 and 52 months), the lack of timely and effective antiviral therapy during pregnancy undoubtedly increased the risk of MTCT. Moreover, despite the fact that both infants who experienced immunization failure were delivered vaginally and there were no cases of immunization failure among infants delivered by cesarean section, there was no statistically significant difference in the impact of these 2 delivery modes on MTCT, as determined by chi-square analysis (Multimedia Appendix 1).

Among the immunized and nonimmunized infants, all positive HBV markers in cord blood, except HBsAb, turned negative at follow-up, except in 2 infants who had HBcAb positivity at the 7-month follow-up. However, the 2 infants who tested positive for HBcAb at follow-up could not be confirmed to have been infected with HBV, as it has been reported that maternal HBcAb can persist in infants for up to 24 months [22].

**Figure 6:**
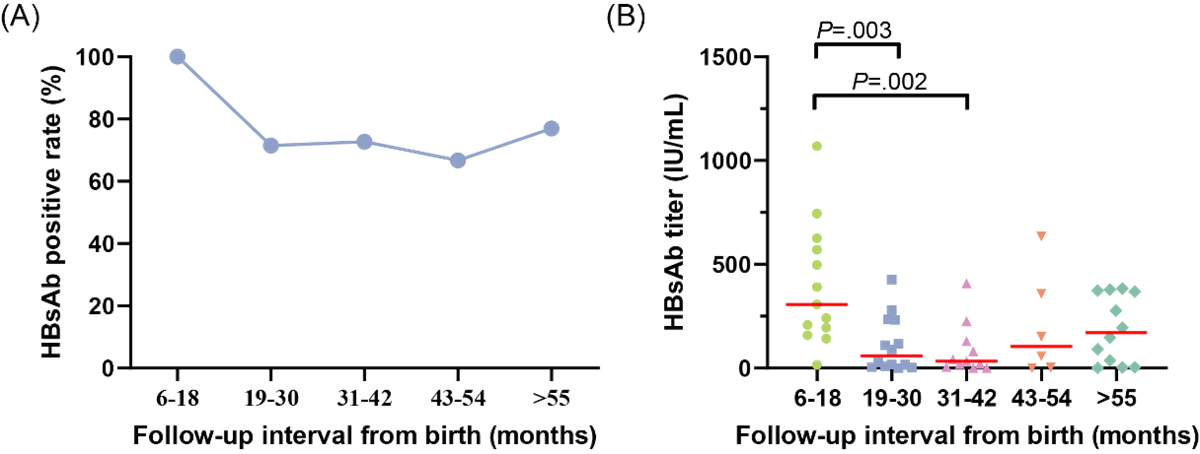
Infants with longer follow-up intervals show a decrease in both the positive rate and titer level of HBsAb compared to the findings in infants with a follow-up interval of 6-18 months. (A) HBsAb-positive rate of infants at different follow-up intervals. (B) The titers of HBsAb in infants at various follow-up intervals (5 results obtained with Roche reagents have been excluded). HBsAb: hepatitis B surface antibody.

To further investigate the duration of HBsAb produced through active-passive immunoprophylaxis, we assessed the positive rate and titer of HBsAb in infants at different follow-up intervals. Moreover, we found that upon timely completion of active-passive immunoprophylaxis, the HBsAb positive rate reached 100% (17/17) among infants followed up at 6-18 months after birth. With the extension of follow-up intervals to 19-30, 31-42, and 42-54 months, there was a declining trend observed in the positive rate of HBsAb, with values of 71% (10/14), 73% (8/11), and 67% (4/6), respectively. However, among infants with a follow-up interval of ≥55 months, there was a slight increase observed in the HBsAb positive rate, which reached 76.9% (Figure 6A).

A comparison was made of the HBsAb titers in 56 infants who were tested at different follow-up intervals using Autobio reagents (excluding 5 results obtained with Roche reagents), as shown in Figure 6B. Similarly, the median HBsAb titers were significantly higher in infants with follow-up intervals ranging from 6 to 18 months than in those with follow-up intervals of 19-30 months (*P*=.003) and 31-42 months (*P*=.002). Furthermore, there was a gradual increase in the median titer of HBsAb when the follow-up intervals were extended to 43-54 months and ≥55 months. Based on our findings during the follow-up, this phenomenon may be attributed to the administration of a booster dose of HepB to certain young children as they approach school entry age.

## Discussion

### Principal Findings

In this study, we observed that the prevalence of hepatitis B among pregnant women in Yinchuan declined from 2017 to 2021, with an overall prevalence rate of 2.53%, which is lower than the reported rate of 3.62% in Ningxia in 2020 [15], indicating the effectiveness of hepatitis B prevention and therapy efforts in Yinchuan. Based on the previous analysis, we can draw several conclusions. First, in the case of implementing neonatal hepatitis B vaccination, a positive HBsAg test with cord blood does not necessarily indicate intrauterine infection and cannot predict the likelihood of immune failure in the neonate. Second, the coverage of antiviral therapy for pregnant women with high viral load in the perinatal period is limited in Yinchuan. Third, it is necessary to strengthen the follow-up of HBV-exposed infants and reduce the failure rate of follow-up. In this regard, we aim to explore the implementation of MTCT prevention methods for hepatitis B.

### Current Strategies for Preventing MTCT in Yinchuan

Numerous studies have explored the underlying factors contributing to the failure of the prevention measures of hepatitis B MTCT. High viral load during pregnancy, the presence of HBeAg in pregnant women, and delayed administration of HBIG and HepB to infants are widely recognized as significant risk factors for MTCT prevention failure [20,23-25]. In response to this situation, China has implemented a series of measures to prevent MTCT, which include performing routine prenatal HBsAg screening for pregnant women, ensuring timely administration of HepB and HBIG to neonates, providing antiviral drugs to pregnant women with high viral loads, and conducting routine follow-up for infants exposed to HBV [14].

In our study, routine prenatal HBsAg screening for pregnant women and the administration of sufficient doses of HepB and HBIG within 24 hours for neonates exposed to HBV have been fully implemented in Yinchuan. Emerging evidence suggests that prompt administration of HBIG following birth can significantly augment the efficacy of combined immunization measures in preventing MTCT of hepatitis B [23,26], particularly when administered within 1 hour after birth [27]. The HBV prevention guidelines have revised the recommended time range for HBIG administration from within 24 hours to 12 hours [18]. The strategy was extensively implemented by YWCHH and its Yuehai branch in 2020.

### Limited Diagnostic Value of HBV Markers in Cord Blood

Infants born to HBV-infected pregnant women may remain susceptible to immune failure and subsequent HBV infection despite receiving vaccinations in practice [28]. A retrospective study indicated that prenatal exposure is not the exclusive cause of MTCT, as intrauterine infection accounts for at least 22% of immune failure cases [29]. Many studies have shown that HBV markers can be transferred from the mother to the fetus through the placenta by cell transfer [25-27]. In the process of delivery, intense uterine contractions lead to the disruption of the placental barrier, resulting in minimal exchange of blood between the mother and placenta. This phenomenon is also considered a potential route for HBV marker transmission [30]. Therefore, some studies have suggested that the presence of HBV markers, such as HBsAg, in cord blood may serve as an indicator for the occurrence of intrauterine infection [31-33]. However, contrasting perspectives exist in other studies, which argue that these HBV markers are transiently positive in infants and can only indicate neonatal exposure to HBV, rather than confirming the occurrence of intrauterine infection [34-36]. The controversy lies in whether the presence of HBV markers in cord blood signifies intrauterine infection.

To investigate the origin of positive HBV markers in cord blood, we conducted a comparative analysis of the concordance rate and titer level variations of corresponding HBV markers in maternal peripheral blood and neonatal cord blood. The findings revealed a significantly higher positive concordance rate of HBeAb and HBcAb between mothers and infants compared to other HBV markers, with a smaller disparity in titer levels between them. This phenomenon may be attributed to differences in the transplacental permeability of HBV markers [34]. HBsAg and HBV DNA have difficulty crossing the placental barrier due to their large spatial structure. Despite its smaller molecular weight and enhanced ability to cross the placental barrier, HBeAg still encounters hindrances. However, as antibodies, both HBeAb and HBcAb can actively transmit to the fetus through the placental barrier. This suggests that the presence of HBV markers in cord blood is more likely attributable to maternal transmission rather than fetal viral replication.

To explore whether the presence of HBsAg or HBV DNA in cord blood can serve as a predictive marker for intrauterine infection and assess its implications for MTCT prevention, we conducted an additional follow-up study on infants who were HBsAg positive in cord blood (among the successfully followed up population, there were 4 individuals with a low viral load). The follow-up results showed that with the timely implementation of complete vaccination measures, almost all positive HBV markers in cord blood, excluding HBsAb, turned negative at the time of follow-up. Meanwhile, 1 infant was diagnosed as having immunization failure at follow-up, despite a negative HBV DNA result in cord blood. A similar pattern has been observed in other studies as well [34,35]. The findings indicate that the presence of HBsAg and HBV DNA in cord blood does not provide conclusive evidence for intrauterine infection or exclude the possibility of neonatal immunization failure [19].

The current hepatitis B immunization policies in China recommend that infants born to mothers who test positive for HBsAg should receive 1 dose of HBIG and 3 doses of HepB free of charge, regardless of cord blood test results. Undoubtedly, this clause undermines the necessity of cord blood testing. Enhancing postnatal monitoring of these infants may prove more advantageous in preventing MTCT.

### Necessity of Expanding the Coverage of Antiviral Therapy for HBsAg-Positive Pregnant Women

Antiviral therapy for pregnant women with a high viral load during the perinatal period to decrease HBV DNA concentration in maternal body fluids that neonates are exposed to during delivery is considered an effective strategy for further decreasing MTCT [4]. In our study, only 12.4% of pregnant women who were positive for HBsAg received antiviral treatment during pregnancy. The findings highlight the urgent need for enhanced coverage of antiviral therapy among pregnant women with a high viral load in Yinchuan. This phenomenon may stem from a lack of awareness among pregnant individuals regarding the efficacy of antiviral therapy, resulting in suboptimal treatment adherence. Between 2014 and 2016, 2 cross-sectional studies were conducted in Guangdong and Shenyang, China, using questionnaires, and they revealed limited levels of awareness and acceptance regarding antiviral treatment among HBsAg-positive pregnant women [37,38]. Although there is no evidence to suggest that the administration of NA drugs, such as TDF, during pregnancy increases the risk of adverse outcomes for both the mother and fetus [39-41], some pregnant women are constrained by outdated stereotypes and decline antiviral therapy due to concerns over potential fetal teratogenic effects [42-44]. Recent survey data indicated a significant increase in the acceptance rate of antiviral treatment among HBsAg-positive pregnant women in Guangdong and Zhejiang provinces [44,45]. The levels of education and awareness regarding hepatitis B among pregnant women are crucial factors influencing the recognition and acceptance of antiviral therapy [38,43-46]. This indicates the imperative for enhanced public education regarding antiviral therapy for hepatitis B, aiming to help pregnant women fully understand its necessity and safety, thus increasing their willingness to cooperate with therapy.

### Necessity of Strengthening Follow-Up for HBV-Exposed Infants

The follow-up of infants born to HBsAg-positive pregnant women still requires further enhancement. In this study, 20% (12/61) of the tracked infants were identified as having failed immunization or having no response to immunization. Previous investigations on the protective efficacy of hepatitis B immunoprophylaxis also revealed similar findings. Infants born to HBsAg-positive mothers exhibited significantly lower titers of HBsAb at a postvaccination serological testing (PVST) program compared to those born to HBsAg-negative mothers [47]. This indicates that fetal exposure to HBV can result in diminished antibody responses among infants. A booster vaccination during adolescence can effectively reduce the seropositivity rate of HBsAg for such individuals [48]. Meanwhile, our findings indicate that infants with longer follow-up intervals are more likely to lose immunity to HBV compared to those who undergo follow-up in the first year after completing vaccination. Long-term follow-up studies on immunity in infants vaccinated against hepatitis B for nearly 2 decades revealed consistent patterns of immune decline [49,50].

The loss of HBV immunity can heighten the vulnerability of infants to infection from HBsAg-positive mothers or other HBV-infected family members [38]. Timely follow-up can validate the efficacy of immunoprophylaxis, ascertain the necessity for booster shots, and identify HBV infection at an early stage. Prompt detection and diagnosis of HBV infection is crucial for subsequent treatment and disease management. Based on these benefits, China proposed a PVST program in 2016, targeting infants born to HBsAg-positive pregnant women, to test for HBsAg and HBsAb, typically 1-2 months following the administration of the third dose of HepB. However, the implementation of the PVST program in other provinces of China has encountered a high failure rate, primarily due to factors such as population mobility, program distrust, limited contact availability, privacy concerns, and parental objection toward the collection of peripheral blood from infants [51-53]. In our study, infant follow-up exhibited a failure rate of 40.8% (42/103), which can be attributed to similar underlying factors. Research indicated that the inclination of individuals to participate in the PVST program was relatively low, particularly among individuals with limited educational attainment. This reluctance can be attributed to the persisting social stigma surrounding HBV infection [52]. To address this dilemma, we recommend further improving the health education for hepatitis B in pregnant women to reduce the psychological stress on HBsAg-positive pregnant women. Moreover, we should further optimize the follow-up procedures to improve the convenience and efficiency of the follow-up. If local economic conditions permit, we will establish incentive mechanisms to reward families who successfully complete the follow-up in order to enhance their enthusiasm for participating in the follow-up.

Overall, the challenges associated with antiviral therapy for pregnant women with a high viral load and the high failure rate during follow-up can primarily be attributed to a lack of understanding regarding hepatitis B, which presents a potential obstacle to the elimination of MTCT for hepatitis B [38,43,54]. The identification of an approach to strengthen the enhancement of public health education on hepatitis B will be the focal point of our forthcoming endeavors.

### Limitations

This study has several limitations. First, the sample of this study was exclusively obtained from a single hospital in Yinchuan, which caters to the largest population of pregnant women. Therefore, the possibility of statistical bias cannot be excluded. In addition, the follow-up population was limited to neonates who tested positive for HBsAg in cord blood, and there was no population composed of neonates who tested negative for HBsAg. Finally, the limited overall sample size at follow-up may have resulted in insufficient statistical power for certain data groups, potentially impacting the validity of the study’s conclusions.

### Conclusion

The findings of this study demonstrate that the prevalence of hepatitis B among local pregnant women in Yinchuan is below the regional average, indicating the effectiveness of local efforts in hepatitis B prevention and control. The clinical significance of HBV marker detection in cord blood is limited under the current prevention measures of MTCT in China. Enhancement of the dissemination of pertinent hepatitis B knowledge among the general public, along with the mitigation of their bias and stigmatization toward hepatitis B, is crucial for effectively implementing measures to prevent MTCT and ultimately attaining the eradication of MTCT related to hepatitis B.

## Appendix

Multimedia Appendix 1 The characteristics of infants under follow-up (N=61).

## Abbreviations

aOR: adjusted odds ratio
COI: cutoff index
HBcAb: hepatitis B core antibody
HBeAb: hepatitis B e-antibody
HBeAg: hepatitis B e-antigen
HBIG: hepatitis B hyperimmune globulin
HBsAb: hepatitis B surface antibody
HBsAg: hepatitis B surface antigen
HBV: hepatitis B virus
HepB: hepatitis B vaccine
LASSO: least absolute shrinkage and selection operator
MTCT: mother-to-child transmission
NA: nucleotide analog
PVST: postvaccination serological testing
TDF: tenofovir disoproxil fumarate
WHO: World Health Organization
YWCHH: Yinchuan Women and Children Healthcare Hospital

## Data Availability

The data associated with this study have not been deposited into a publicly available repository. The data will be made available upon reasonable request.
