# Supplementary material for: Investigation of Mother-to-Child Transmission of Hepatitis B in Yinchuan, China: Cross-Sectional Survey Study
Source: JMIR Public Health Surveill. 2024 Sep 4;10:e60021. doi: 10.2196/60021 (PMC11411227; doi:10.2196/60021)
Supplement: Multimedia Appendix 1 [file publichealth_v10i1e60021_app1.doc]

**Multimedia Appendix 1.** The characteristics of infants under follow-up (N=61).

| Characteristics | | HBsAg (+) | HBsAg (-) | χ² | *P* value |
| --- | --- | --- | --- | --- | --- |
|  |  | (n=2) | (n=59) |  |  |
| **Neonatal gender, n (%)** | |  |  | N/A^a^ | >.99 |
|  | Male | 1 (50) | 31 (53) |  |  |
|  | Female | 1 (50) | 28 (47) |  |  |
| **Neonate birth weight, n (%)** | |  |  | 0.951 | >.99 |
|  | Normal | 0 (0.00) | 2 (3) |  |  |
|  | Low (<2500 g) | 2 (100) | 53 (90) |  |  |
|  | Excessive (>4000 g) | 0 (0) | 4 (7) |  |  |
| **Preterm birth (weeks <37), n (%)** | |  |  | N/A | >.99 |
|  | Yes | 0 (0) | 3 (5) |  |  |
|  | No | 2 (100) | 56 (95) |  |  |
| **Delivery method, n (%)** | |  |  | N/A | .52 |
|  | Vaginal | 2 (100) | 27 (46) |  |  |
|  | Caesarean | 0 (0) | 17 (29) |  |  |
|  | Not recorded | 0 (0) | 15 (25) |  |  |
| **Maternal age, n (%)** | |  |  | 1.39 | .98 |
|  | 18-34 | 1 (50) | 29 (49) |  |  |
|  | ≥35 | 1 (50) | 30 (51) |  |  |
| **Maternal HBeAg status, n (%)** | |  |  | N/A | .18 |
|  | Positive | 2 (100) | 24 (41) |  |  |
|  | Negative | 0 (0) | 35 (59) |  |  |
| **Maternal HBV DNA load, n (%)** | |  |  | 3.064 | .20 |
|  | High viral load | 1 (50) | 6 (10) |  |  |
|  | Low viral load | 0 (0) | 12 (20) |  |  |
|  | Negative | 0 (0) | 16 (27) |  |  |
|  | Not tested | 1 (50) | 25 (42) |  |  |
| **Infant HBeAg status at birth, n (%)** | |  |  | N/A | .13 |
|  | Positive | 2 (100) | 20 (34) |  |  |
|  | Negative | 0 (0) | 39 (66) |  |  |
| **Infant HBV DNA load at birth, n (%)** | |  |  | N/A | .21 |
|  | Low viral load | 1 (50) | 5 (8) |  |  |
|  | Negative | 1 (50) | 48 (81) |  |  |
|  | Not tested | 0 (0) | 6 (10) |  |  |

^a^ N/A: not applicable.
